# Prevention of fall incidents in patients with a high risk of falling: design of a randomised controlled trial with an economic evaluation of the effect of multidisciplinary transmural care

**DOI:** 10.1186/1471-2318-7-15

**Published:** 2007-07-02

**Authors:** Geeske MEE Peeters, Oscar J de Vries, Petra JM Elders, Saskia MF Pluijm, Lex M Bouter, Paul Lips

**Affiliations:** 1 Institute for Research in Extramural Medicine, VU University Medical Center, Amsterdam, The Netherlands; 2 Department of Internal Medicine, section of Geriatrics, VU University Medical Center, Amsterdam, The Netherlands; 3 De Grote Rivieren, Primary Care Practice, Amsterdam, The Netherlands; 4 Department of Public Health, Erasmus MC, Rotterdam, The Netherlands; 5 Department of Endocrinology, VU University Medical Center, Amsterdam, The Netherlands

## Abstract

**Background:**

Annually, about 30% of the persons of 65 years and older falls at least once and 15% falls at least twice. Falls often result in serious injuries, such as fractures. Therefore, the prevention of accidental falls is necessary. The aim is to describe the design of a study that evaluates the efficacy and cost-effectiveness of a multidisciplinary assessment and treatment of multiple fall risk factors in independently living older persons with a high risk of falling.

**Methods/Design:**

The study is designed as a randomised controlled trial (RCT) with an economic evaluation. Independently living persons of 65 years and older who recently experienced a fall are interviewed in their homes and screened for risk of recurrent falling using a validated fall risk profile. Persons at low risk of recurrent falling are excluded from the RCT. Persons who have a high risk of recurrent falling are blindly randomised into an intervention (n = 100) or usual care (n = 100) group. The intervention consists of a multidisciplinary assessment and treatment of multifactorial fall risk factors. The transmural multidisciplinary appraoch entails close cooperation between geriatrician, primary care physician, physical therapist and occupational therapist and can be extended with other specialists if relevant. A fall calendar is used to record falls during one year of follow-up. Primary outcomes are time to first and second falls. Three, six and twelve months after the home visit, questionnaires for economic evaluation are completed. After one year, during a second home visit, the secondary outcome measures are reassessed and the adherence to the interventions is evaluated. Data will be analysed according to the intention-to-treat principle and also an on-treatment analysis will be performed.

**Discussion:**

Strengths of this study are the selection of persons at high risk of recurrent falling followed by a multidisciplinary intervention, its transmural character and the evaluation of adherence. If proven effective, implementation of our multidisciplinary assessment followed by treatment of fall risk factors will reduce the incidence of falls.

**Trial registration:**

Current Controlled Trials ISRCTN11546541.

## Background

Fall incidents are the third cause of chronic disablement in older persons according to the WHO [1]. Annually, about 30% of persons older than 65 years falls at least once and 15% falls at least twice [2-4]. The consequences of falling are severe: 5% of the falls leads to a fracture and 5% of the falls leads to other serious injuries [5,6]. About one in four fallers consults a hospital emergency room or primary care physician after the fall [6]. These facts emphasize the necessity of measures to prevent falling in older persons.

The pathogenesis of falling is multifactorial [2,7]. Causes of falling are impairments in balance, gait, muscle strength, visual acuity and cognition, chronic diseases and use of psychotropic medication [8-12]. Many studies have investigated risk factors of falling [2,13-15] and several risk profiles have been developed [4,14,16-18], which can be used to identify older persons with a high risk of falling.

Interventions to reduce the risk of falling have been successful to a varying degree. Home visits by nurses were found to be ineffective [19], whereas Tai Chi, exercise therapy and multifactorial interventions led to a decrease in falls [20-22]. A meta-analysis showed that a multifactorial fall risk assessment and management programme was effective in all older populations investigated, both with high or low risk of falling [23]. A systematic Cochrane review of preventive interventions showed a positive effect in older persons with a history of falling or in those who were known to have risk factors [24].

The guideline "Prevention of fall incidents in older persons", developed by the Dutch Institute for Healthcare Improvement (CBO), recommends a systematic assessment of fall risk factors in independently living older persons with a high risk of falling. Based on this assessment, a specific and individual treatment plan has been designed [25]. A similar strategy has previously been investigated in the Prevention Of Falls in the Elderly Trial (PROFET) study in the UK, leading to a fall incidence reduction of 50% [13]. However, other studies that evaluated the effectiveness of multifactorial fall prevention strategies were not effective [26-31]. Only one trial studied the cost-effectiveness of a multifactorial intervention program in the USA and reported that the intervention was associated with fewer falls and lower costs [32]. Although many geriatric outpatient clinics have recently started "multidisciplinary fall prevention services", no studies have yet been conducted assessing the effectiveness and cost-effectiveness of such a multifactorial intervention program in older persons with a high risk of recurrent falling.

The objective of this article is to describe the design of a randomised controlled trial that aims to reduce the fall risk in older persons with a high risk of falling. The intervention consists of a systematic assessment of the putative causes of falling and subsequent targeted individualised preventive measures. Unique characteristics of this trial are the evaluation of fall risk factors and subsequent treatment of persons with a high risk of recurrent falling, and the close collaboration between the hospital and primary care physician (transmural care). Both the effectiveness and cost-effectiveness of the intervention will be assessed.

## Method

### Study design and randomisation

**Figure 1:**
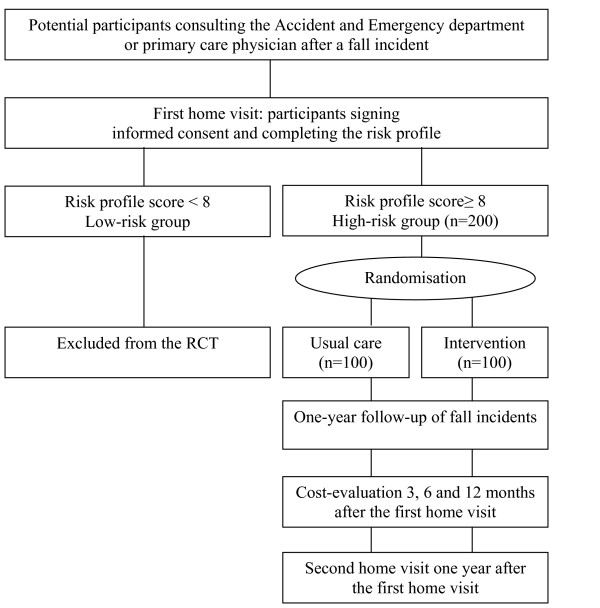
Design of the study.

This study is a randomised controlled trial (RCT) with a one-year prospective follow-up. Simultaneously, an economic evaluation will be conducted. The Medical Ethics Committee of the VU University Medical Center has approved the study design, protocols and informed consent procedures. Figure 1 shows the design of the study. Potential participants are contacted and after signing informed consent a validated fall risk profile [16] is used to select participants with a high risk of recurrent falling (score of 8 and higher). Participants with a low-risk of recurrent falling are excluded from the RCT. Participants living in a residential home are right away assigned to the high-risk group, which is in accordance with the recommendations of the Dutch Institute for Healthcare Improvement (CBO) guideline [25]. Participants in the high-risk group are blindly randomised into two groups: the intervention group and the usual care group. Prior to the onset of the trial a randomisation schedule is made by a statistician. A block randomisation of 4 per block is used. Using the sequence of this schedule, opaque envelopes are numbered and filled with group names. When a participant is designated to the high-risk group, the interviewer, who is unaware of the content, opens the envelope with the lowest number.

### Study population

The study population consists of persons of 65 years and over, who consult the Accidents and Emergency Department of the VU University Medical Center (Amsterdam, The Netherlands) or their primary care physician between April 2005 and April 2007 after a fall incident. Inclusion criteria are living independently or in a residential home, living in the vicinity of the VU University Medical Center and having experienced a fall. Exclusion criteria are inability to sign informed consent, inability to provide a fall history, fall due to a traffic or occupational accident, living in a nursing home and acute pathology requiring long-term rehabilitation, such as a stroke.

200 high-risk participants will be included in the intervention study, of which we will enrol 100 participants in the intervention group and 100 in the control group. With a significance level of 0.05, a power of 80% and an expected difference in fall incidence of 50% [13] between the intervention and control group, 57 participants are needed both in the intervention and in the control group. Taking into account a drop-out rate of 30% a minimum of 82 participants are needed in each group. With 100 persons in both the intervention and control group, the numbers are certainly high enough to detect statistical significant differences.

### Procedure

All persons who consult the emergency department or primary care physician after a fall receive usual care. Within two weeks after the initial presentation, written information is sent and several days thereafter, the potential participants are contacted by telephone. All actual participants (who sign informed consent) are visited at their homes by a trained interviewer within 3 months after the fall incident. During the first home visit, the fall risk profile [16], fall history, independence in activities of daily living, quality of life and physical performance are measured. All high-risk participants will report falls during at least one year using a fall calendar and receive a cost-evaluation questionnaire at 3, 6 and 12 months after the first home visit. The times to first and second fall are the primary outcome measures. One year after the first home visit, they are visited a second time to reassess the activities of daily living, quality of life and physical performance. Scores on these questionnaires and tests are used as secondary outcome measures. Furthermore, the treatment adherence and medication use are evaluated. Table 1 presents an overview of the procedure and measurements. The measurements used are described later on in this article.

**Table 1:** Overview of procedure and measurements

	Months	Measurements	Purpose
1st home visit	0	fall risk profile	selection high risk participants
		fall history	assessment of fall risk factors, confounders
		activities of daily living	secondary outcome measure
		quality of life	secondary outcome measure
		medication use	assessment of fall risk factors
		medical history	assessment of fall risk factors, confounders
		physical performance	secondary outcome measure
1st follow-up	3	1st calendar sheet	primary outcome measure
		3 months cost-evaluation	primary outcome measure
2nd follow-up	6	2nd calendar sheet	primary outcome measure
		6 months cost-evaluation	primary outcome measure
3rd follow-up	9	3rd calendar sheet	primary outcome measure
2nd home visit	12	activities of daily living	secondary outcome measure
		quality of life	secondary outcome measure
		medication use	treatment adherence
		medical history	secondary outcome measure, confounders
		physical performance	secondary outcome measure
		treatment adherence	confounder, to enhance the interpretation of the results
4th follow-up	12	4th calendar sheet	primary outcome measure
		12 months cost-evaluation	primary outcome measure

### Intervention

An extended multidisciplinary assessment starts with a visit to the geriatric outpatient clinic. A multifactorial fall risk assessment will be conducted aiming to identify modifiable fall risk factors. The assessment of fall risk factors and design of the treatment plan is based on the directives in the CBO guideline "Prevention of fall incidents in older persons" [25]. The assessment consists of a general medical and drug history, a fall and mobility history and physical examination. According to the recommendations of the CBO guideline special emphasis is placed on signs and symptoms of potentially modifiable fall and fracture risk factors such as postural hypotension, visual impairment, parkinsonism, osteoporosis, osteoarthritis, gait disorders, psychotropic and cardiac drug use, and environmental hazards. When indicated, additional diagnostic tests can be performed (e.g. laboratory tests or imaging). Based on the assessment of risk factors an individually tailored treatment regimen aimed at reduction of the fall risk is composed in close collaboration with the general practitioner of the participant. In the Netherlands, the general practitioner has a central role in the coordination of specialist's care, home care and physiotherapy among others. The collaboration facilitates the transmural continuity of care that has been lacking in most previously performed fall risk reduction trials. The multifactorial treatment can consist of, for example, withdrawal of psychotropic drugs, balance and strength exercises by a physical therapist, home hazard reduction by an occupational therapist or referral to an ophthalmologist or cardiologist. Per participant and per diagnosis the International Classification of Diseases code (ICD10) code and recommendations are reported to document the treatment regimen.

### Usual care

Usual care in The Netherlands after a fall mainly consists of treatment of the consequences of the fall. Although the CBO guideline was released in 2004 [25], multifactorial fall risk prevention has not yet been implemented by general practitioners or at the accidents and emergency departments. In primary care settings 'usual care' only incidentally includes systematic assessment and treatment of fall risk factors.

### Measurements

#### Baseline assessment

During the first home visit, the risk of recurrent falling, fall history, medical history, medication use, independence in activities of daily living and quality of life are assessed. Risk of falling is assessed using a fall risk profile [16]. This profile, which was developed and validated in the Longitudinal Aging Study Amsterdam, [16] is used to screen participants with a high risk of recurrent falling. Recurrent falling is defined as 2 or more falls in a 6-month period [8,16,18]. This profile consists of 5 questions, 2 measurements (handgrip strength and body weight) and 2 interaction items. To measure handgrip strength a digital strain-gauged dynamometer (Takei TKK 5401, Takei Scientific Instruments Co. Ltd., Tokyo, Japan) is used. To measure body weight, a calibrated balance beam scale is used. On each item, points are scored and the scores are summed (range 0–30). Table 2 presents the diagnostic values of the risk profile for different cut off points on the fall risk score for 426 participants in the LASA-study who reported at least one fall in the previous year [16]. For this study, participants are defined at high risk of recurrent falling as the total score is 8 or higher. For an optimal combination of sensitivity and specificity, a cut-off score of 11 should be used. However, with a cut-off score of 11, a low sensitivity is obtained and too many participants with a high risk of recurrent falling will be missed. To ensure that not too many low risk participants are falsely diagnosed as high risk, the specificity should not be too low. At a cut-off score of 8, the sensitivity is higher than 50% and the specificity is higher than 70%.

**Table 2:** Diagnostic values of the risk profile at different cut-off points in the total risk score

Cut-off in the total risk score	% at high risk group	Sensitivity (%)	Specificity (%)	Σ^a ^(%)	PV+ (%)	PV-	P_falls_
0 vs. ≥ 1	782	95.1	11.7	106.8	41.1	78.8	0.10 vs. 0.34
0–1 vs. ≥ 2	69.7	86.1	22.1	108.2	41.8	71.0	0.11 vs. 0.36
0–2 vs. ≥ 3	61.5	79.9	33.8	113.7	43.9	72.1	0.13 vs. 0.39
0–3 vs. ≥ 4	51.4	70.8	47.3	118.1	46.6	71.4	0.14 vs. 0.43
0–4 vs. ≥ 5	46.0	66.0	54.5	120.5	48.5	71.2	0.15 vs. 0.46
0–5 vs. ≥ 6	39.2	60.4	64.0	124.4	52.1	71.4	0.17 vs. 0.50
0–6 vs. ≥ 7	35.4	55.6	68.0	123.6	53.0	70.2	0.17 vs. 0.52
0–7 vs. ≥ 8	32.6	52.1	71.2	123.3	54.0	69.6	0.18 vs. 0.54
0–8 vs. ≥ 9	29.8	48.6	74.3	122.9	55.1	69.0	0.19 vs. 0.56
0–9 vs. ≥ 10	27.9	46.5	76.6	123.1	56.3	68.8	0.20 vs. 0.57
0–10 vs. ≥ 11	21.4	41.0	85.6	126.6*	64.8	69.1	0.22 vs. 0.91
0–15 vs. ≥ 16	5.9	13.9	97.7	111.6	80.0	63.6	0.28 vs. 0.77

^a ^sum of sensitivity + specificity; * maximum Σ; PV+ positive predictive value; PV- negative predictive value; P_falls _probability of recurrent falls in low risk versus high risk group. The sample used in LASA to develop the fall risk profile included relatively healthy community dwelling older persons of which a large part reported zero previous falls [16]. In contrast, all participants of this study have a history of at least one recent fall and also include people living in a home for the elderly. Thus, the participants in our study are expected to have poorer mobility and more functional limitations and, on average, to score higher on the risk profile. The diagnostic values presented here are the recalculated values using the data of 426 independently living participants of the LASA study who fell at least once [16].

Fall history is assessed with the fall history instrument (Carefall Triage Instrument, version 007), which is a questionnaire developed by the Academic Medical Center, the VU University Medical Center and the Erasmus Medical Center, The Netherlands. The fall history collects data on the circumstances of the last and previous falls, mobility and risk factors of bone loss and osteoporosis, social status and general health. Medical history is assessed using a questionnaire on self-reported (chronic) diseases both in the past and present. The questionnaire includes 7 major chronic diseases, i.e. chronic lung diseases, cardiac diseases, vascular diseases, stroke, diabetes mellitus, malignant neoplasms and joint disorders (i.e. osteoarthritis and rheumatoid arthritis). In addition, participants are asked to indicate any other chronic disease, including psychological diseases, which they have or have had. Medication use is assessed by directly copying the names of drugs used in the previous two weeks from the containers. The name, doses per unit, number of times taken per day, time of administration, doses per intake, purpose, prescription or over the counter (OTC) and whether the drug has been prescribed after the fall are assessed for each drug. This information is compared to medication records of the public pharmacy. Level of independence in activities of daily living (ADL) will be examined using the Barthel Index (range 0, fully dependent, to 20, fully independent) [33]. The level of functioning on more complex daily activities will be examined using a scale of instrumental ADL introduced by Lawton and Brody (range 0, fully dependent, to 8, fully independent) [34]. Quality of Life (QoL) is examined using the Dutch translations of the SF-12 and the EQ-5D. The SF-12 consists of 12 items and is a abbreviated version of the SF-36 [35]. The SF-12 has been designed and validated to measure health related QoL in large population studies [35,36]. The EQ-5D (EuroQol) has been developed to generate a general index of experienced health and for the assessment of Quality Adjusted Life Years (QALY). It is therefore suitable for economic evaluations [37]. The assessment consists of 5 items (mobility, self-care, usual activities, pain/discomfort and anxiety/depression) and a visual analogue scale (0, worst, to 100, best imaginable health state) [37]. The fall history, ADL and QoL questionnaires are sent to the participants prior to the first home visit. Participants are asked to complete these questionnaires before the home visit. The visiting researcher assists participants who are unable to complete these questionnaires independently.

**Figure 2:**
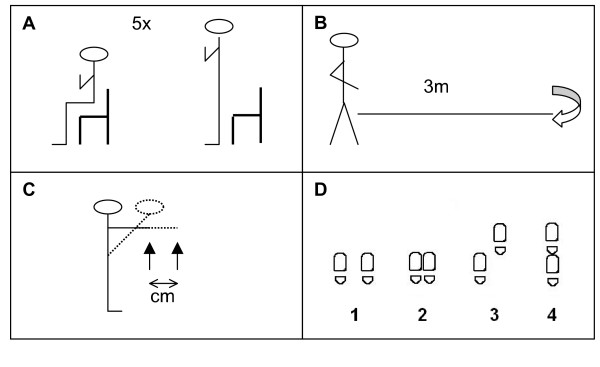
Physical performance tests. **A **Chair stands test. **B **Walk test. **C **Functional reach. **D **Modified Romberg test.

To assess physical performance, four tests are conducted (see Figure 2). The chair stands test is a standardized test in which the participant stands up and sits down for five consecutive times as fast as possible with the arms folded in front of the chest [38]. During the walk test the participant walks 3 meter along a line, turns 180 degrees and walks 3 meters back along the line [38]. Time is recorded in both tests from start to finish. The functional reach is a standardized test to measure the ability to maintain balance while reaching forward [39]. The participant stands parallel to a wall with one arm horizontally stretched and then leans forward while keeping the arm stretched and horizontal. The distance between the start and end position of the index finger is measured in centimetres. The modified Romberg test is used as a measure for standing balance. The participant stands consecutively with the feet apart at shoulder width, with the feet side to side, with one foot in front of the other but not in one line and with the feet in one line and heel against toe (tandem stand). All positions are performed first with the eyes open and then with the eyes closed. The participant scores 1 point per position continued for at least 10 seconds (range 0, poor balance, to 4, good balance) [40].

#### Follow-up

At the first home visit the participants receive a fall calendar [16]. For the period of one year, the participants tick per week whether they did or did not fall during that week. A fall is defined as an unintentional change in position resulting in coming to rest at a lower level or on the ground [41]. Once per 3 months the participants return a calendar sheet by mail. When no sheet is received, or when the sheet is completed incorrectly, we inquire by telephone whether, and if yes, when the participant has fallen in the past 3 months.

The cost-evaluation questionnaire registers the costs made to prevent a new fall or the consequences of a new fall. The questionnaire assesses: the number of visits to physicians, therapists or day care centres; amount and aim of surgical procedures; number of days of admission to a hospital, home or nursing home; purchase of aids and adaptations in the home environment. The questionnaires are conducted 3, 6 and 12 months after the first home visit. The 3 and 6 months questionnaires are sent by mail and apply to the preceding 3 months. The 12 months questionnaire is assessed during the interview of the second home visit. This questionnaire applies to the preceding 6 months. When no questionnaire is received, or when it is completed incorrectly, the questionnaire will be completed by telephone.

During the second home visit, one year after the first visit, the same physical performance tests are conducted along with a reassessment of fall related healthcare costs, medical history, medication use, quality of life and activities of daily living. In addition, in the intervention group, adherence to the treatment regimen is evaluated. Treatment adherence in the intervention group is evaluated per recommendation given. Recommendations regarding changes in medication are evaluated by reassessing the medication use as described above. Adherence to all other recommendations (such as referrals to physical therapy or cardiologist) is assessed by asking whether, to what extent, and how the recommendations of the intervention were effected. The information from the participant is completed with information from the rapports of the involved specialists.

### Statistics

Data will be primarily analysed according to the intention-to-treat principle, i.e., including all randomized participants, regardless of whether they received or did not receive the intervention. Subsequently, the results of the intention-to-treat analysis will be compared with the results of an on-treatment analysis.

At baseline, differences in baseline characteristics will be compared between the intervention and control group to examine comparability between the two groups.

To examine the effectiveness of the multidisciplinary transmural care, Cox proportional hazards regression will be conducted, with time to first fall and time to second fall within one year of follow-up as outcome measures, and with age, gender and living situation as covariables. Subsequently, multiple linear regression analyses will be used to compare differences in secondary outcome measures (ADL-score, QoL, physical performance and morbidity data) at 12 months follow-up between intervention and control group.

The economic evaluation will be conducted from a societal perspective, which implies that all costs and outcomes are taken into account. The economic evaluation will involve calculating cost-effectiveness and cost-utility ratios. The incremental costs and effects of the intervention will be compared with usual care. The difference in costs of the intervention group and usual care group will be computed using bootstrapping techniques. Uncertainty ratios will be presented on cost effectiveness and cost utility planes. Acceptability curves will also be estimated. These present the probability that the intervention is cost-effective given a ceiling ratio that policy makers are willing to invest.

### Progress of the study

In April 2005 the inclusion of the participants started and will continue until July 2007. The follow-up will end in July 2008 and then data-analysis will be initiated.

## Discussion

The strengths of this study, are the screening of participants at high risk of falling, its transmural design and evaluation of treatment adherence. In previous studies [13,42], as in our study, participants were selected at accident and emergency departments or primary care centres. These participants are a mix of once-fallers and recurrent fallers. In previous studies, the participants assigned to the intervention group were not selected for risk of falling. We expect that participants at high risk of recurrent falling will benefit most from the intervention and, therefore, we expect to find an enhanced (cost-) effectiveness of the preventive measures.

Furthermore, in previous studies, the recommendations of the intervention were drawn up by the geriatrician and executed by the primary care physician [13,42], whereas in this study, the primary care physician is actively involved in the process of drawing up the recommendations, and the intervention is initiated by the geriatrician and followed up by both the geriatrician and primary care physician. We therefore expect to have a better coordination of the transmural care.

In several fall prevention studies, active participation has been associated with better outcomes [43,44] and poor treatment adherence has been reported as a possible explanation for lack of effect of the intervention [45]. In contrast to previous studies [13,42,43], we not only score the number of recommendations that are effected, but also add an evaluation of how the treatment recommendations are effected. This information will add to the interpretation of the results [44].

The results of this trial will provide clinicians in the field of aging with more knowledge on treatment of older persons at high-risk of recurrent falling. If proven cost-effective, a multidisciplinary assessment and treatment of fall risk factors in persons with a high risk of recurrent falling will lower the risk of falling and consequently lead to reduced incidence and costs of falls.

## Competing interests

The author(s) declare that they have no competing interests.

## Authors' contributions

GP and OV are responsible for collection and processing of the data. GP drafted the manuscript. All authors contributed to the conception and design of the study. All authors read and corrected draft versions of the manuscript and have given approval of the printed version.

## Pre-publication history

The pre-publication history for this paper can be accessed here:


